# Supplementary figures and images for: Bioactive Potential of Two Marine Picocyanobacteria Belonging to Cyanobium and Synechococcus Genera
Source: Microorganisms. 2021 Sep 28;9(10):2048. doi: 10.3390/microorganisms9102048 (PMC8537962; doi:10.3390/microorganisms9102048)

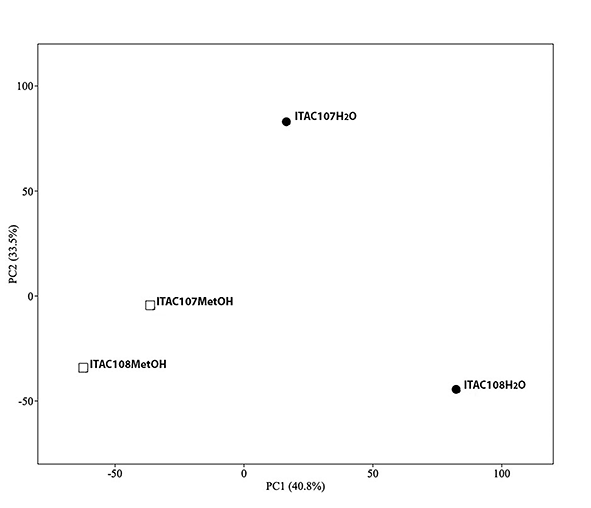

Supplement: Supplementary file 1 [file microorganisms-09-02048-s001.zip › microorganisms-1393779-supplementary/FigureS1.tif]
